# The Effect of Rilonacept versus Placebo on Health-Related Quality of Life in Patients with Poorly Controlled Familial Mediterranean Fever

**DOI:** 10.1155/2014/854842

**Published:** 2014-05-15

**Authors:** Philip J. Hashkes, Steven J. Spalding, Rula Hajj-Ali, Edward H. Giannini, Anne Johnson, Karyl S. Barron, Michael H. Weisman, Noune Pashinian, Andreas O. Reiff, Jonathan Samuels, Dowain Wright, Daniel J. Lovell, Bin Huang

**Affiliations:** ^1^Cleveland Clinic Foundation, 9500 Euclid Avenue, Cleveland, OH 44195, USA; ^2^Pediatric Rheumatology Unit, Shaare Zedek Medical Center, P.O. Box 3235, 91031 Jerusalem, Israel; ^3^Cincinnati Children's Hospital Medical Center, 3333 Burnet Avenue, Cincinnati, OH 45229, USA; ^4^Division of Intramural Research, National Institute of Allergy and Infectious Diseases, National Institutes of Health, DHHS, 33 North Drive, MSC 3207, Bethesda, MD 20892, USA; ^5^Cedars-Sinai Medical Center, 8700 Beverly Boulevard, Los Angeles, CA 90048, USA; ^6^Children's Hospital of Los Angeles, 4650 Sunset Boulevard, Los Angeles, CA 90027, USA; ^7^NYU Langone Hospital for Joint Diseases, 301 East 17th Street, New York, NY 10003, USA; ^8^Children's Hospital Central California, 9300 Valley Children's Place, Madera, CA 93636, USA

## Abstract

*Objective*. To examine the effect of rilonacept on the health-related quality of life (HRQoL) in patients with poorly controlled familial Mediterranean fever (FMF). *Methods*. As part of a randomized, double-blinded trial comparing rilonacept and placebo for the treatment of FMF, patients/parents completed the modified Child Health Questionnaire (CHQ) at baseline, and at the start and end of each of 4 treatment courses, 2 each with rilonacept and placebo. *Results*. Fourteen subjects were randomized; mean age was 24.4 ± 11.8 years. At baseline the physical HRQoL score was significantly less (24.2 ± 49.5) but the psychosocial score was similar to the population norm (49.5 ± 10.0). There were significant improvements in most HRQoL concepts after rilonacept but not placebo. Significant differences between rilonacept and placebo were found in the physical (33.7 ± 16.4 versus 23.7 ± 14.5, *P* = 0.021) but not psychosocial scores (51.4 ± 10.3 versus 49.8 ± 12.4, *P* = 0.42). The physical HRQoL was significantly impacted by the treatment effect and patient global assessment. *Conclusion*. Treatment with rilonacept had a beneficial effect on the physical HRQoL in patients with poorly controlled FMF and was also significantly related to the patient global assessment. This trial is registered with ClinicalTrials.gov Identifier NCT00582907.

## 1. Introduction

Familial Mediterranean fever (FMF) is a genetic autoinflammatory disorder caused by mutations in the chromosome 16* MEFV* gene, encoding the protein pyrin. Patients experience recurrent episodes of fever, serositis, arthritis, and rash with late complications of renal amyloidosis in untreated patients [1–3]. 5–10% of patients develop chronic arthritis. Clinical manifestations start before age 10 and 20 years in 80% and 90% of the patients, respectively. Attacks with varying intensities usually continue throughout life. FMF occurs mainly in Armenians, Arabs, Jews, and Turks and is estimated to have a worldwide prevalence of 100,000 to 150,000 patients [4].

Treatment with colchicine is effective in reducing the frequency of attacks in most patients and prevents the development of amyloidosis in nearly all patients [5–8]. However, 30–40% of patients are only partially responsive and 5–10% are nonresponsive or intolerant of colchicine [9, 10].

Since FMF is a lifelong episodic disease, it is logical that many aspects of the health-related quality of life (HRQoL) may be affected, especially in patients with frequent attacks. Indeed, several studies have found that the HRQoL of patients with FMF is decreased, particularly the physical aspects [11–17]. In part this may be related to a higher prevalence of FMF patients with fibromyalgia, anxiety, and depression than the general population [11, 17–19]. However, these studies were of cross-sectional design conducted on convenience patient cohorts and none examined the effect of treatment in a longitudinal manner.

We recently concluded a controlled study showing that rilonacept (Regeneron, Tarrytown, NY, USA), an interleukin (IL)-1 fusion protein decoy receptor [20], is effective in decreasing the number of attacks (the primary outcome) in patients with poorly controlled FMF when compared to placebo [21]. One of the secondary objectives of the study was to compare the effect of rilonacept and placebo on the HRQoL. We found that only the physical but not psychosocial aspect of HRQoL was decreased at baseline when compared to the general population. Treatment with rilonacept had a positive effect only on the physical aspects of HRQoL which was significantly better than when participants received placebo. However the scope of the primary manuscript did not enable us to report in detail on HRQoL which was one of many secondary outcomes defined a priori.

HRQoL is a key concern for patients with this lifelong disease and for treating physicians. Therefore, we expand on HRQoL in this report with the following aims.

1. To describe individual concepts composing HRQoL in severely affected FMF patients, showing which concepts are especially affected by this disease.
2. To describe the changes from screening/baseline after treatment with rilonacept and placebo and report differences between the treatment groups.
3. To identify demographic, clinical, laboratory, and treatment factors that significantly influence HRQoL.

## 2. Patients and Methods

### 2.1. Subjects and Study Design

The study design was previously described in detail [21]. In brief, this was a multicenter, randomized, double-blind, single-subject, and alternating treatment study. After a one-month screening phase to determine eligibility and the frequency of FMF attacks, the 14 participants were randomized to 1 of 4 treatment sequences that included 2 three-month treatment courses with subcutaneous injections of rilonacept (2.2 mg/kg/week, max 160 mg) and 2 with placebo. Colchicine was continued at the participants' prestudy dose. Participants who developed ≥2 attacks during any treatment course (rilonacept or placebo) were allowed to “escape” to the other treatment arm until the end of that course (blinding was maintained) and then resume their assigned sequence. The Institutional Review Boards at all sites approved the study protocol. Understanding and informed consent were obtained from all adult subjects or parents/legal guardians for subjects <18 years.

### 2.2. Health-Related Quality of Life Assessments

Study participants or in the case of children parents/guardians completed the English language version of the 50-item Child Health Questionnaire (CHQ) Parent Form-50. While validated (in more than 20 languages) in children as a parent administrated questionnaire [22, 23], the wording of the questions was minimally adapted for this study to allow adult participants to answer the same questions as in the validated version. Examples of modifications included wording changes of* your child* to* you/your child*,* school* to* work/school*, and so forth.

The 50 CHQ items are incorporated into 15 separate concepts including physical function (PF), role/social limitations due to physical health (RP), bodily pain and discomfort (BP), general health perceptions (GH), role/social limitations due to emotional or behavioral difficulties (REB), behavior (BE), mental health (MH), self-esteem (SE), emotional impact on parent/patient (PE), time impact on parent/patient (PT), global health (GGH), global behavior (GBE), change in health (CH), family activities (FA), and family cohesion (FC). Higher scores indicate better HRQoL. Raw scores for all concepts except CH were transformed to a 0–100 scale (CH remained a categorical score on a scale of 1–5). In addition two summary scores, the physical summary score (PhS) and the psychosocial summary score (PsS) were calculated based on weighted scores of 10 from the 15 concepts. For the PhS, 4 of the concepts related more to physical health were given a positive weight (PF, RP, BP, and GH), while 6 related more to psychosocial assessments were weighted negatively. For the PsS, 6 concepts related more to psychosocial assessments (REB, BE, MH, SE, PE, and PT) were weighted positively while the 4 related more to physical health were weighted negatively. Summary scores were standardized by the normal population to a mean score of 50 and standard deviation of 10.

### 2.3. Data Collection and Statistical Analysis

The CHQ was administered at the screening and baseline visits, at the start and end of each treatment course, and at the time of escape visits. Thus, subjects who completed the entire study answered the CHQ between 6 and 8 times (no participants had more than 2 escape visits).

Descriptive statistics, including means and confidence intervals, were provided for scores at screening, baseline, and start of treatment courses. Individual and groups scores were compared between placebo and rilonacept courses and changes were calculated between treatment courses and screening and baseline scores. The study outcomes were comparisons of PhS and PsS standardized scores. Additional analyses included the scores of individual concepts. Since PhS and PsS reflect two distinct measures of HRQoL, no multiple comparison test was performed. Given the very small sample size, no multiple comparison tests were performed for individual CHQ concepts.

CHQ scores were averaged over placebo and rilonacept courses. We used mixed model analyses that took into account random effects between and within participants, with treatment courses as the within participant factor. Using the intent to treat (ITT) principle, all randomized patients were analyzed. To be consistent with the primary paper the main analyses included data only prior to escape [21]. Sensitivity analyses including data after escape visits were performed, with the escape factor considered in the mixed model.

To identify bivariate associations between potential factors and HRQoL outcomes, we first conducted correlation analyses for continuous variables and analysis of variance (ANOVA) for categorical variables. Determinants included baseline demographic data as well as clinical and laboratory data from baseline and following treatment courses. Clinical determinants included the frequency and length of attacks, the proportion of time patients experienced an attack, characteristics of attacks (including degree of fever and other systems involved), the patient/parental and physician global assessment assessed by marking a Likert-like 0 (best) –10 (worst) scale and the Modified Armenian Severity Score [24]. Laboratory determinants included acute phase reactants (erythrocyte sedimentation rate (ESR), C-reactive protein (CRP), fibrinogen and serum amyloid A (SAA), and platelet levels). Variables showing Spearman correlations stronger than 0.45 or −0.45 or with a *P* value <0.05 in either the physical or psychosocial summary scores were then incorporated into mixed model analyses. Two models were performed; one forcing and one without forcing the treatment effect into the model. The models examined the influence of these determinants and the treatment group on the physical and psychosocial HRQoL outcomes.

Missing data were treated as missing at random, consistent with the primary analyses paper. All analyses were conducted using SAS 9.3 (SAS Institute Inc., Cary, NC, USA).

## 3. Results

The study included 14 participants (Table 1). All participants completed the CHQ at screening and baseline. For 11 participants who completed the 12-month study the CHQ was completed after all rilonacept and placebo courses (except missing data in 2 participants from 1 rilonacept course each), 10 rilonacept escape courses from 7 participants, and 4 placebo escape courses from 3 participants. One participant (a nonresponder) who left the study after 2 treatment courses completed the CHQ after 1 rilonacept, 1 placebo, 1 rilonacept escape, and 1 placebo escape course. Two participants completed the CHQ only after the 1st treatment course (1 placebo and 1 rilonacept).

**Figure 1:**
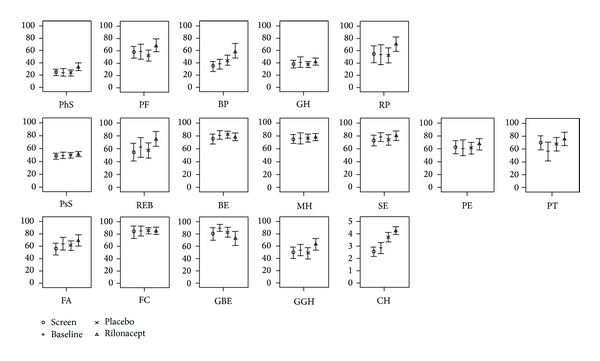
Health-related quality of life scores measured at screening and baseline visits and after treatment with rilonacept and placebo. Results are reported as a mean and 95% confidence interval. The 1st row includes the physical summary score and the 4 individual concepts weighted positively in calculating this score. The 2nd row includes the psychosocial summary score and the 6 concepts weighted positively in calculating this score. The 3rd row includes those concepts not used in calculating the summary scores. PhS: physical summary score; PF: physical function; BP: bodily pain and discomfort; GH: general health perceptions; RP: role/social limitations due to physical health; PsS: psychosocial summary score; REB: role/social limitations due to emotional or behavioral difficulties; BE: behavior; MH: mental health; SE: self-esteem; PE: emotional impact on parent/patient; PT: parental/patient time impact; FA: family activities; FC: family cohesion; GBE: global behavior; GGH: global health; CH: change in health.

**Figure 2:**
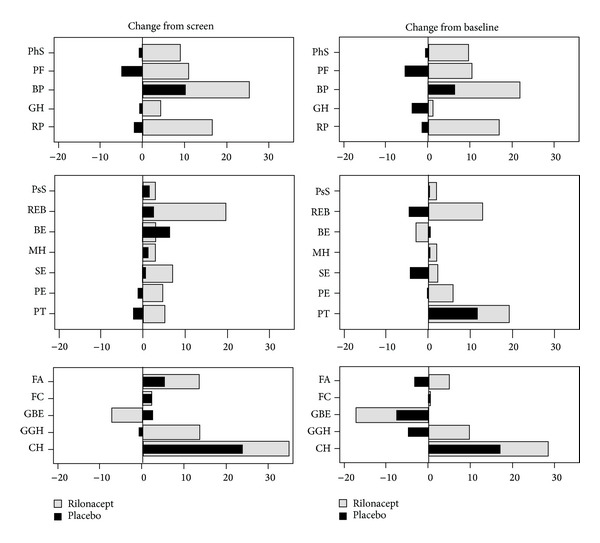
Changes in health-related quality of life scores after use of rilonacept or placebo when compared to the screening and baseline visits. The order of the concepts is the same as Figure 1. CH was multiplied by 20 to align score with other concepts. PhS: physical summary score; PF: physical function; BP: bodily pain and discomfort; GH: general health perceptions; RP: role/social limitations due to physical health; PsS: psychosocial summary score; REB: role/social limitations due to emotional or behavioral difficulties; BE: behavior; MH: mental health; SE: self-esteem; PE: emotional impact on parent/patient; PT: parental/patient time impact; FA: family activities; FC: family cohesion; GBE: global behavior; GGH: global health; CH: change in health.

The mean and corresponding 95% confidence interval of HRQoL concepts and summary scores at screening, baseline, during treatment with rilonacept and placebo are shown in Figure 1. The most affected HRQoL concepts were bodily pain (BP) and general health (GH). As a result the PhS was markedly decreased at screening and baseline (Figure 1).  Figure 2 shows changes in HRQoL concepts and summary scores during rilonacept and placebo treatment compared to screening and baseline. There was improvement in all HRQoL concepts during rilonacept treatment compared to screening except for global behavior (GBE) and for GBE and behavior (BE) compared to baseline visits (Figure 2). Marked improvements of more than 10 points (in scale of 0–100) were seen in 7 concepts compared to screening and 6 concepts compared to baseline visits (Figure 2). On the other hand 6 and 9 concepts worsened when receiving placebo compared to screening and baseline visits, respectively (Figure 2). Marked improvement of more than 10 points was seen only in change in health (CH) compared with both screening and baseline visits and with bodily pain (BP) compared to screening and time impact (PT) compared to baseline visits.

The PhS improved by 8 and 9 points (0–100 scale, normalized to population) while receiving rilonacept when compared to screening and baseline visits, respectively, while there was much less improvement in the PsS (3 and 2 points, resp.,). During placebo there was a minimal worsening in the PhS and a minimal improvement in the PsS compared to both screening and baseline visits (Figures 1 and 2). Thus, there was a significant difference between rilonacept and placebo in the PhS (*P* = 0.025, by signed rank test). However, even after treatment with rilonacept the PhS was still below the population norm of 50 (Table 2(a)).

Analyses of changes of individual concepts of HRQoL showed significant differences between rilonacept and placebo only in physical function (PF), with near significant differences in 3 other concepts (Tables 2(a) and 2(b)). Sensitivity analyses including post-escape data was similar to the primary analysis.

### 3.1. Determents of Health-Related Quality of Life

Correlation analyses found that the attack frequency and proportion of time patients experienced an attack, patient/parent global assessment and modified Armenian severity score all had a correlation <−0.45 (with *P* < 0.03) with the PhS while proportion of time patients experienced an attack patient/parent global assessment, age (worse in adults) and ethnicity showed significant correlations with the PsS (Table 3). Abnormal ESR and fibrinogen levels were significantly associated with a worse PhS while older age was significantly associated with a worse PsS. The mixed model found that the global patient assessment (*P* < 0.0001) and treatment arm (*P* = 0.047) were significant determinates of the PhS while only the proportion of days in attack (*P* = 0.003) was a significant determinant of the PsS.

## 4. Discussion

These data show that the decrease in HRQoL in FMF patients with poorly controlled disease was mainly in the physical aspects, especially in physical function and global health and to a lesser degree in bodily pain. However, psychosocial aspects of HRQoL were near the population norm at baseline prior to study intervention, despite having prolonged disease with a mean disease duration >17 years.

Several studies have shown that the HRQoL of FMF patients and their families is decreased [11–17]. Similar to our results, Deger et al., using the Short-Form (SF-) 36 in adults, found significant differences between 90 FMF patients and 67 controls only in the physical but not mental component summary [14]. In that study patients with anxiety and depression also had a decrease in the mental component scores when compared to controls. Also Giese et al. who examined 40 Turkish and 40 German patients with FMF and 40 controls by the World Health Organization Quality of Life scale (WHOQOL-BREF) found decreased HRQoL only in physical concepts [16]. This was in contrast to 2 studies of 51 children and adolescents and 100 adults with FMF that demonstrated decreased physical and emotional HRQoL measured by the Pediatric QoL Inventory Generic Core Questionnaire and SF-36, respectively [13, 15]. Makay et al. found that school function, not examined in the CHQ, was the most important QoL concept affected in children with FMF [13].

There is a debate whether there is a correlation between the frequency of attacks and HRQoL with Buskila et al. and Makay et al. finding a significant correlation with the attack frequency [11, 13], while Sahin et al. and Giese et al. did not [15, 16]. Since all patients in our study had very frequent attacks (nearly one per week in the screening month), it was not surprising that we did not find this to be a significant factor. Buskila et al. found that HRQoL was worse in older patients and those with longer disease duration [11]. We also found that the psychosocial HRQoL was worse in older patients.

The association of FMF with fibromyalgia, found in 1.8–32% of FMF patients [11, 17–19], may be an important contributor to a poor HRQoL [11, 17]. It is important to stress that none of the participants in our study had fibromyalgia, having only episodic symptoms, although we did not use screening instruments to specifically exclude fibromyalgia.

HRQoL was examined during a treatment trial of the cryopyrin-associated periodic syndromes (CAPS) using canakinumab, an IL-1*β* antibody [25]. Similar to FMF, CAPS are also episodic monogenic autoinflammatory diseases. Marked improvement was seen in the HRQoL, especially in bodily pain and physical concepts, as in our study. However, unlike our study the improvement reached the population norm. Two major differences between the studies were the much lower baseline PhS in our study compared to the CAPS study (24.2 versus 43.4). Also 1/3 of the participants in our study were nonresponders versus only 3% in the CAPS study. In absolute numbers the increase in the PhS was similar in both studies (9-10 points on a scale of 0–100). In other FMF studies, Ozçakar et al. also found a significant improvement in daily activity, weakness, and appetite as well as sleep quality in 50 children with FMF after treatment with colchicine was started [26]. In cross-sectional studies, Makay et al. and Sahin et al. showed that partial or nonresponders to colchicine had worse HRQoL than complete responders [13, 15].

Improving HRQoL may have an impact on the FMF attack frequency in addition to the direct effect of medications. Stressors (good and bad) are one of the important triggers of FMF attacks [27]. In a study of 45 children with FMF, psychosocial factors, particularly the child's hostility, contributed to 27% of the variability in attack frequency [28]. A retrospective study of 11 FMF colchicine resistant patients treated with selective serotonin reuptake inhibitors for depression (3 patients also had fibromyalgia) showed a >95% decrease in the frequency of attacks before and after this treatment was added to colchicine as well as a significant decrease in inflammatory markers [29].

Study limitations are obviously the small sample size of 14 subjects and the related limitation of analyzing multiple variables that may affect HRQoL. However, this was the first study to report on the HRQoL of colchicine resistant patients and to include HRQoL as an a priori outcome measure. Another limitation is the use of the HRQoL CHQ tool also in adults, though developed primarily for children and adolescents [22]. Since this small study included all age groups, we preferred, for simplicity, to use one questionnaire that can be easily adapted for all age groups with minor changes. A HRQoL study of children and adults with severe CAPS similarly used the CHQ [30]. Most of the questions and concepts in the CHQ are very similar or even identical to the widely used adult SF-36 and are scored in similar manner with a summary physical and psychosocial score. Furthermore, our main comparisons were within individual participants before and after treatment and not with the general population. It is also possible our results are applicable to severely affected, but not to the general FMF population.

## 5. Conclusions

We demonstrated that patients with longstanding FMF (without fibromyalgia) poorly controlled with colchicine had significantly lower physical-, but not psychosocial-, related HRQoL, which significantly improved after treatment with rilonacept, but not with placebo. Improving the HRQoL is an important patient driven outcome with this lifelong disease in addition to other outcomes traditionally used such as reducing attack frequency and inflammatory markers and should be one of the primary treatment objectives in treating FMF.

## Figures and Tables

**Table 1:** Basic data on study participants (*N* = 14).

Age at enrollment (yrs), mean (SD), and range	24.4 (11.8), 4.5–47.4
Gender	8 (57%) male; 6 (43%) female
Age at diagnosis (yrs), mean (SD), and range	6.8 (5.7), 2–25
Disease duration (yrs), mean (SD), and range	17.5 (12.6), 0.7–43.7
∗Frequency of attacks per screening month	3.3 (1.2, 3.6), 1–4.6
∗Frequency of attacks per month of rilonacept	0.8 (0.2, 1.2), 0–5
∗Frequency of attacks per month of placebo	2 (0.9, 2.4), 0.5–3.1

*Attack frequency data is shown by medians (1st and 3rd quartiles), ranges.

**(a):** 

HRQoL concept	∗Rilonacept mean (SD)	∗Placebo mean (SD)	Rilonacept-placebo differences (SD)	*P* value
**PhS**	**33.66 (16.41)**	**23.7 (14.51)**	**9.58 (3.69)**	**0.025**
PF	68.18 (28.54)	52.31 (26)	16.01 (6.45)	0.031
BP	59.55 (31.99)	44.17 (23.39)	14.61 (6.81)	0.055
GH	42.12 (16.52)	37.26 (13.85)	3.84 (3.54)	0.30
RP	70.45 (30.83)	52.08 (35.21)	17.09 (8.64)	0.074

*Higher score is better (score of 0–100).

PhS: physical summary score; PF: physical function; BP: bodily pain and discomfort; GH: general health perceptions; RP: role/social limitations due to physical health.

**(b):** 

HRQoL concept	∗Rilonacept mean (SD)	∗Placebo mean (SD)	Rilonacept-placebo differences (SD)	*P* value
**PsS**	**51.4 (10.31)**	**49.79 (12.37)**	**1.4 (2.27)**	**0.55**
REB	74.75 (30.9)	57.41 (33.84)	15.25 (8.58)	0.10
BE	78.37 (16.13)	81.74 (15.05)	−3.57 (3.35)	0.31
MH	77.95 (14.45)	76.3 (19.14)	1.15 (2.88)	0.70
SE	80.11 (20.25)	73.61 (22.54)	7.32 (4.19)	0.11
PE	67.05 (23.07)	61.11 (26.43)	5.96 (5.64)	0.31
PT	74.75 (28.31)	67.13 (29.76)	7.35 (7.69)	0.36

*Higher score is better (score of 0–100).

PsS: psychosocial summary score; REB: role/social limitations due to emotional or behavioral difficulties; BE: behavior; MH: mental health; SE: self-esteem; PE: emotional impact on parent/patient; PT: parental/patient time impact.

**Table 3:** Associations between demographic, clinical and laboratory variables of familial Mediterranean fever rilonacept study participants and physical/psychosocial health-related quality of life.

	Physical score	Psychosocial score
Correlation coefficients		
Attack frequency	*r* = −0.46 *P* = 0.021	*r* = −0.30 *P* = 0.14
Proportion of days in attack	*r* = −0.68 *P* = 0.0002	*r* = −0.73 *P* < 0.0001
Patient global assessment	*r* = −0.75 *P* < 0.0001	*r* = −0.56 *P* = 0.003
Physician global assessment	*r* = −0.56 *P* = ** 0.003**	*r* = −0.46 *P* = 0.017
Modified Armenian severity score [24]	*r* = −0.54 *P* = ** 0.005**	*r* = −0.24 *P* = 0.24
Low grade fever (37-38)	*r* = −0.10 *P* = 0.63	*r* = 0.06 *P* = 0.76
Medium grade fever (38-39)	*r* = −0.02 *P* = 0.93	*r* = −0.36 *P* = 0.07
High grade fever (>39)	*r* = −0.15 *P* = 0.47	*r* = −0.10 *P* = 0.61
Abdominal pain	*r* = −0.21 *P* = 0.29	*r* = −0.02 *P* = 0.93
Chest pain	*r* = 0.06 *P* = 0.75	*r* = −0.06 *P* = 0.76
Musculoskeletal symptoms	*r* = 0.21 *P* = 0.30	*r* = 0.13 *P* = 0.51

Analysis of variance (ANOVA) tests		
^*∧*^Age	*P* = 0.45	*P* = 0.022
^ #^Ethnicity	*P* = 0.33	*P* = 0.02
^†^Erythrocyte sedimentation rate	*P* = 0.010	*P* = 0.96
^†^C-reactive protein	*P* = 0.49	*P* = 0.67
^†^Serum Amyloid A	*P* = 0.23	*P* = 0.84
^†^Fibrinogen	*P* = 0.049	*P* = 0.78
^†^Platelets	*P* = 0.17	*P* = 0.65

Continuous variables were analyzed by Spearman's correlation and categorical variables by analysis of variance.

Significant results are bolded.

^∧^Age groups included >18 (*N* = 10) and ≤18 years (*N* = 4); ^#^Ethnicity groups included Arab Christians (*N* = 4), Armenians (*N* = 5), and others (*N* = 5); ^†^Acute phase reactants were dichotomized for analysis as either normal or abnormal.
